# Better Cognitive Performance Is Associated With the Combination of High Trait Mindfulness and Low Trait Anxiety

**DOI:** 10.3389/fpsyg.2018.00627

**Published:** 2018-05-03

**Authors:** Satish Jaiswal, Shao-Yang Tsai, Chi-Hung Juan, Wei-Kuang Liang, Neil G. Muggleton

**Affiliations:** ^1^Institute of Cognitive Neuroscience, National Central University, Taoyuan City, Taiwan; ^2^Institute of Cognitive Neuroscience, University College London, London, United Kingdom; ^3^Department of Psychology, Goldsmiths, University of London, London, United Kingdom

**Keywords:** personality traits, mindfulness, anxiety, executive functions, self-report measures

## Abstract

There are several ways in which cognitive and neurophysiological parameters have been consistently used to explain the variability in cognitive ability between people. However, little has been done to explore how such cognitive abilities are influenced by differences in personality traits. Dispositional mindfulness and anxiety are two inversely linked traits that have been independently attributed to a range of cognitive functions. The current study investigated these two traits in combination along with measures of the attentional network, cognitive inhibition, and visual working memory (VWM) capacity. A total of 392 prospective participants were screened to select two experimental groups each of 30 healthy young adults, with one having high mindfulness and low anxiety (HMLA) and the second having low mindfulness and high anxiety (LMHA). The groups performed an attentional network task, a color Stroop task, and a change detection test of VWM capacity. Results showed that the HMLA group was more accurate than the LMHA group on the Stroop and change detection tasks. Additionally, the HMLA group was more sensitive in detecting changes and had a higher WMC than the LMHA group. This research adds to the literature that has investigated mindfulness and anxiety independently with a comprehensive investigation of the effects of these two traits in conjunction on executive function.

## Introduction

Differences in personality traits have a consequential influence on cognitive abilities and quality of life. Most previous studies have investigated the impact of traits on cognitive functions by employing subjective self-report measures (Dobson, 2000; Walsh et al., 2009; Rammstedt et al., 2016). Nevertheless, some recent studies have explored how such variability in traits might affect higher order executive functions, which can be defined as a set of regulatory mechanisms necessary for general-purpose control of behavior (Miyake and Friedman, 2012), such as the efficiency of the attentional network (Pacheco-Unguetti et al., 2010; Tanaka et al., 2013; Di Francesco et al., 2017), conflict monitoring (Bishop, 2009), and VWM capacity (Moriya and Sugiura, 2012). Dispositional mindfulness and anxiety are two predominant traits that have largely been studied independently and shown to have an impact on several cognitive tasks (Dobson, 2000; Pacheco-Unguetti et al., 2010; Quickel et al., 2014; Di Francesco et al., 2017). Mindfulness can be regarded as a naturally occurring capacity which facilitates attending to the experiences of the present moment in a non-judgmental way (Kabat-Zinn, 1990; Brown and Ryan, 2003; Bishop et al., 2004). Anxiety, in comparison, has been described as an emotion that negatively affects top–down processing in executive functions and enhances susceptibility toward irrelevant salient stimuli (Eysenck and Calvo, 1992; Eysenck et al., 2007; Moser et al., 2012).

There are two correlational self-report studies which seem to suggest an inverse relationship between mindfulness and anxiety, in one case in a non-clinical population (Coffey and Hartman, 2008) and in one case in a clinical group (Desrosiers et al., 2013). Both studies seem to suggest that mindfulness may be mediated through different emotion regulation strategies such as worry, reappraisal, non-acceptance, and rumination with these strategies acting like a ‘buffer’ for anxiety levels and it is suggested that this might result in attenuation of the psychopathology associated with anxiety (Coffey and Hartman, 2008; Desrosiers et al., 2013). In other words, any impairment in mindfulness capacity may lead to malfunctioning in emotion regulation pathways and may lead to anxiety, although it should be remembered that anxiety as an emotion and emotion regulation are two different constructs (Cisler and Olatunji, 2012).

Anxiety can be characterized as a defensive behavior in response to a potential threat and may bring about sympathetic arousal affecting physiology, behavior, and cognition. This is mainly governed by the amygdala (Davis, 1992; Barlow, 2004; Lipp, 2006). Anxiety can impair cognitive efficiency even in the absence of threat stimuli, with one explanation of this offered by the attentional control theory (Eysenck et al., 2007; Berggren and Derakshan, 2013). Emotion regulation is related to effort to change the emotional state in order to employ approaches meant to generate explicit behavioral and cognitive responses and is principally controlled by the PFC and functionally-linked surrounding brain areas (Quirk et al., 2006; Quirk and Mueller, 2008). A large body of literature suggests that functional connectivity between PFC and amygdala facilitates emotion regulation (Davidson, 2002; Creswell et al., 2007; Frewen et al., 2010) and is involved in monitoring impairments in performance due to threat-induced anxiety (Davidson, 2002; Gold et al., 2015). There are several studies that have shown that PFC also plays a critical role in executive functions (e.g., Miyake et al., 2000; Tang et al., 2010; Teper and Inzlicht, 2013). The above studies suggest that executive function and emotion regulation share common or interconnected brain regions. There is also very limited empirical evidence about how differences in levels of mindfulness, which also reportedly modulate emotion regulation strategies (Desrosiers et al., 2013), may alter cognitive performance.

The emotion regulatory model (Coffey and Hartman, 2008; Desrosiers et al., 2013), which suggests a mediating interaction between mindfulness and anxiety, has been strengthened by two electrophysiological reports that indicated trait mindfulness (Brown et al., 2013) and trait anxiety (Mocaiber et al., 2009) could predict variability in the amplitude of the late positive potential (LPP) electroencephalographic event-related potential component, an objective index of an emotional response. Brown and colleagues found that individuals with high mindfulness scores showed smaller LPP amplitudes in response to high arousal unpleasant stimuli than did individuals with low mindfulness, whereas Mocaiber et al. (2009) observed high trait anxiety individuals had higher LPP amplitudes than did low trait anxiety individuals. Thus, a smaller amplitude of LPP as a function of trait mindfulness (Brown et al., 2013) and higher amplitude of LPP among individuals with trait anxiety (Mocaiber et al., 2009) seems to suggest an inverse relationship between these two traits. However, this does not necessarily demonstrate any reciprocal relationship. Moreover, the link between dispositional mindfulness and trait anxiety through emotion regulation is supported by two neuroimaging studies (Etkin et al., 2004; Way et al., 2010). In the study by Etkin et al. (2004), a higher level of trait anxiety was predictive of elevated amygdala activity, while Way et al. (2010) observed that higher mindfulness was associated with lower amygdala activity.

Considering the above mediational, electrophysiological, and fMRI evidence leads the current research to follow the proposal that mindfulness and anxiety may be mediated by the emotion regulation system (worry, reappraisal, non-acceptance, and rumination) through which they may interact in an antagonistic manner (Coffey and Hartman, 2008; Greeson and Brantley, 2009). However, it cannot necessarily be generalized that the amygdala is the only neural substrate facilitating any resilience or beneficial effect relating to elevated mindfulness or a lowered anxiety level, as it is functionally heterogeneous in nature, and can be activated by many other factors (Díaz-Morán et al., 2013). Broad trait constructs such as mindfulness and anxiety have often been reported to interact with other brain areas such as the PFC (Creswell et al., 2007; Bishop, 2009), anterior cingulate cortex (Tang et al., 2010; Klumpp et al., 2012), and the posterior parietal lobe (Berryhill and Olson, 2008), areas that have roles in a range of executive functions such as selective attention, conflict control and WMC amongst others (Miyake et al., 2000; Gallant, 2016).

There are several studies on executive functions that have explored how trait mindfulness (e.g., Di Francesco et al., 2017) or trait anxiety (e.g., Bishop, 2009; Pacheco-Unguetti et al., 2010; Moriya, 2016) independently affect the performance of cognitive tasks. However, there is a lack of behavioral studies that might further establish the nature of any association between the two traits. Hence, there are potential limitations in studies which account for effects of either dispositional mindfulness or anxiety independently.

The current study is an attempt to explore the nature of the association between the two traits by proposing that dispositional mindfulness and trait anxiety are usually, but not necessarily always, closely linked and investigate the combined impact on three major domains of executive functions when they are linked: conflict monitoring, inhibition, and working memory. Given the evidence for a frequent link between mindfulness and anxiety, defining groups based on a combination of the two factors should (1) provide groups with distinct or non-overlapping characteristics, so giving a better idea of the cognitive functions that are affected by mindfulness/anxiety and (2) allow comparison with previous findings where groups were defined on just one of these factors. From previous correlational studies (Coffey and Hartman, 2008; Desrosiers et al., 2013), it was expected that individuals with HMLA or LMHA would be more common than individuals with high mindfulness, high anxiety or low mindfulness, low anxiety in a normal sample of the population. Therefore, we proposed that comparing the two groups of participants would be more informative about the seemingly common factor underlying both HMLA (or the opposite).

Pertaining to the impact of personality traits on behavioral performance, Di Francesco et al. (2017) observed that the modulation of three networks of attention (Fan et al., 2002) could be predicted by different facets of mindfulness, whereas in a study on trait anxiety Pacheco-Unguetti et al. (2010) observed that only executive networks seemed to be modulated by trait anxiety, with there being no effect on alerting and orienting networks. In addition to differences in constructs of mindfulness and anxiety, it is also possible that these differences in behavioral performance are due to definition of the groups investigated in relation to a single trait (i.e., mindfulness or anxiety). It would be beneficial to investigate how these attentional networks are modulated by mindfulness and anxiety in conjunction. In addition, neither of these reports evaluated the secondary performance measure of the ANT task (Fan et al., 2002), which is accuracy rate. A recent cross-sectional (Jo et al., 2016) study that employed ANT did not observe any group differences (between meditators and control) on three attentional networks, but did see that the meditator group accuracy was higher than the control group. The current study also aimed to investigate this additional measure of performance on the ANT even though accuracy rate is a more general measure of attention (Prinzmetal et al., 2005) controlled by motor areas (Heitz and Schall, 2012; Tarrasch et al., 2017) rather than specific executive control which is mostly governed by prefrontal areas (Kane and Engle, 2002).

It has also been observed that there are very few studies which have looked at how cognitive inhibition is affected by either mindfulness or anxiety, either independently or in conjunction (Moore and Malinowski, 2009). In their cross-sectional meditation research, Moore and Malinowski (2009) observed that irrespective of whether a participant was a meditator or a control participant, their error rate on a CST (Stroop, 1935) negatively correlated with global mindfulness scores, although meditators showed a lower error rate than the control group. The color Stroop paradigm does not require processing of any affective information during task performance, but still can test cognitive conflict. This makes this task suitable to measure the structure and stability of cognitive control independent of circumstances. As suggested by Eysenck et al. (2007), if a non-threatening stimulus can act as an effective distractor (i.e., the color words in the CST), it is possible to better generalize the theory under investigation from findings from such a task. Thus, the current study employed a CST to measure the cognitive inhibition aspect of executive control, in addition to accuracy measure for general attention.

Several studies have looked into how mindfulness interventions enhance WMC (Jha et al., 2010; Mrazek et al., 2013). Although there are some studies which have looked into how anxiety may affect WMC (Darke, 1988; Moriya and Sugiura, 2012) there is a limited number of studies which have looked at how WMC can be influenced by trait mindfulness (Vines, 2014). In the first of these, Vines (2014) did not observe any effect of dispositional mindfulness on WMC. Although Darke (1988) reported highly anxious people showed a lower WMC, this contrasts with the study by Moriya and Sugiura (2012) that found that highly socially anxious people showed a higher WMC.

These differences in findings might be due the fact that either these studies employed different paradigms [an operation span task in the case of Darke (1988) and a change detection task in the case of Moriya and Sugiura (2012)] or different questionnaires to measure anxiety. The operation span task (Unsworth et al., 2005) provides a measure of verbal working memory whereas the change detection task (Luck and Vogel, 1997) estimates visuo-spatial WMC. Nevertheless, the possibility of the difference in the above findings being due to differences in paradigms could be overruled, as anxiety has been suggested to negatively affect both constructs (verbal and visual) WMC (Moreno et al., 2015; Moran, 2016). Differences in the constructs of anxiety being investigated in both these studies could be a primary reason for differences in observations. The study by Moriya and Sugiura (2012) employed a social anxiety questionnaire (in this case the STAI-State) (Spielberger et al., 1970) was used as measure of social anxiety) while Darke (1988) administered the Test Anxiety Scale (Wine, 1971). Moriya and Sugiura (2012) argued that socially anxious individuals could have high VWM capacity. However, according to the attentional control theory (Eysenck et al., 2007), anxiety impairs the processing of target relevant information and therefore results in limited WMC, in line with a number of previous studies (see Moran, 2016, for a review). Taking the above findings into account, the present study employed the STAI-Trait (Spielberger et al., 1970) questionnaire to measure the level of trait anxiety. Moreover, the current study mainly focused on the visual and spatial domains of attention as reflected by the tasks chosen above, so employed a change detection task rather than an operation span task to further assess the relationship between mindfulness/anxiety and WMC.

Performance on three cognitive tasks, the ANT (Fan et al., 2002), the CST (Stroop, 1935) and a change detection task (Luck and Vogel, 1997), was investigated to assess how two combined traits (mindfulness and anxiety) may affect three important domains of executive function: conflict monitoring, cognitive inhibition, and WMC respectively (Miyake et al., 2000; Fan et al., 2002). As suggested by Miyake et al. (2000), executive functions, such as the ability to maintain target information over time (WMC) with simultaneous selective attention to competing information in the environment (conflict control), plays a critical role in the completion of complex higher order cognitive tasks. Furthermore, it has been demonstrated that dorsolateral PFC plays a critical role in maintenance of WMC, as well-having a role in selective attention, consistent with WMC and selective attention having a structural as well as a functional connection (see Kane and Engle, 2002, for a review). Additionally, another previous study (Long and Prat, 2002) showed that high WMC individuals showed a strong Stroop effect only when the incongruent stimuli were infrequent. Low WMC individuals, in contrast, showed a stronger Stroop interference effect that was not related to the proportion of these trials. Selective attention and Stroop interference seem to both interact with WMC. Hence, the ANT, CST, and change detection tasks were employed in the current study to test the efficiency of the selective attentional network system, cognitive inhibition, and WMC, respectively.

A range of cross-sectional and longitudinal meditation research has reported meditators having a higher level of mindfulness and that this may facilitate behavior as well as aspects of brain plasticity (Tang et al., 2007, 2012; Manna et al., 2010; Baijal et al., 2011; Srinivasan and Singh, 2017). There is also convergent evidence from meditation training and cross-sectional studies showing that meditators show reduced symptoms of anxiety compared to control groups or conditions (Kabat-Zinn et al., 1992; Hofmann et al., 2010). It should be noted, however, that individuals with varying degree of mindfulness or anxiety and meditators are distinct populations.

Based on previous reports from meditation research and assumptions made above, we aimed to primarily look at cognitive differences, if any, associated with trait levels of mindfulness and anxiety in conjunction. It was tested whether individuals selected on the basis of objectively defined selection criteria based on the two inversely correlated personality traits of mindfulness and anxiety showed different behavioral performance. This current study is correlational in design, comparing differences in executive functions between the two groups (HMLA and LMHA). As such, there is no experimental manipulation, neither with respect to mindfulness nor to anxiety, as it mainly focuses on exploration of the implications of mindfulness and anxiety as trait measures. In addition, sex as a factor was evaluated merely to check for invariance. The hypotheses investigated were that individuals with HMLA would perform better on the ANT (Tang et al., 2007; Jo et al., 2016), a CST (Moore and Malinowski, 2009; Teper and Inzlicht, 2013) and on a VWM change detection task (Jha et al., 2010; Zeidan et al., 2010) than those with LMHA. It was also predicted that for the ANT the HMLA group would show faster reaction times and higher accuracy rates than the LMHA group, and for the CST, the HMLA group would show smaller differences in reaction times (i.e., less of a Stroop effect) and higher accuracy rates than the LMHA group. Similarly, for the change detection task, the HMLA group was predicted to show higher accuracy rates, higher sensitivity in detecting changes and a higher WMC than the LMHA group.

## Materials and Methods

### Participants

An advertisement for recruitment was placed on a Facebook group page (NCU TALK) of National Central University, Taiwan, between July 2016 and December 2016. The prospective participants (*N* = 392) filled in two online questionnaires; the Chinese versions of the MAAS (Brown and Ryan, 2003; Chang et al., 2015) and the STAI-Trait (Spielberger et al., 1970; Shek, 1993). There were some exclusion criteria for participants such as a requirement that they should not have any prior experience of any style of meditation (mindfulness, Zen, Tai Chi, Qi Gong, and Yoga). There was also an age limit with a range from 18 to 30 years old. Potential participants undergoing psychiatric treatment, pharmacological treatment, or with any neurological disease were excluded. The selection procedure for the participants is described below.

### Double Selection Criteria

The participants were categorized by employing a mean ± standard deviation criterion rather than using median split analysis (MacCallum et al., 2002). It has been argued that it is inappropriate to consider that values just above or below the median, as would be the case for many individuals when a median split categorization is used, are meaningfully different from each other. Additionally, it can be quite difficult to delineate an effect that already exists using such a split (Aiken et al., 1991; McClelland et al., 2015). For instance, there are two independent studies on trait mindfulness in which one reported that dispositional mindfulness could successfully predict emotional reactivity (Brown et al., 2013), while other reported that it could not (Cosme and Wiens, 2015). Therefore, in the current study we adopted a mean ± SD criteria to categorize individuals into groups according to mindfulness and anxiety levels.

A total of 392 people (215 females) responded to the advertisements and completed both questionnaires mentioned above. The theoretical range (minimum–maximum) for mindfulness scores was 15–90 and for trait-anxiety scores was 20–80. The average mindfulness score for the entire sample pool was 61.6 with SD of 10.6, while the mean trait anxiety was 46.6 with a SD of 9.2. There were no overall sex differences for trait mindfulness scores (males: 62.2 ± 10.2; females: 61.6 ± 11.0; *t* = 0.548; *p* = 0.584) or for trait anxiety scores (males: 46.7 ± 9.5; females: 46.2 ± 9.0; *t* = 0.526; *p* = 0.599). There was a significant negative correlation between the two questionnaire scores (*r* = -0.490, *p* < 0.0001). The subjects were selected for the HMLA if their MAAS scores (α = 0.860) were greater than or equal to 70 (mean + 0.85 SD) and STAI-Trait scores (α = 0.880) were less than or equal to 39 (mean: 0.85 SD). If their MAAS scores were less than or equal to 53 (mean: 0.85 SD) and their STAI-T scores greater than or equal to 54 (mean + 0.85 SD) they were assigned to the LMHA. The other combinations of these measures gave high mindfulness and high anxiety (HMHA) individuals and low mindfulness and low anxiety (LMLA) individuals, with the rest of the prospective participants lying in an ‘intermediate’ group. This resulted in the following numbers of participants: HMLA (46 individuals), HMHA (9 individuals), LMLA (3 individuals), LMHA (38 individuals), and intermediate (296) (**Table 1**). As expected, the numbers of HMHA and LMLA groups were too low to be statistically compared to the groups for the HMLA and LMHA. Hence in the current study only two groups, the HMLA group and the LMHA group, were investigated for cross-sectional differences in behavioral performance, with the distributions of genders balanced for each group.

**Table 1:** Summary of distribution of prospective participants in the sample pool.

Categories in sample pool	Mindfulness cut off scores	Anxiety cut off scores	Number of individuals	% of total population	Age (in years)	Group mindfulness Scores	Group anxiety scores
High mindfulness and high anxiety	≥70	≥54	9 (5F)	2.3	20.8 ± 0.7	73.1 ± 2.2	56.1 ± 2.7
High mindfulness and low anxiety^∗^	≥70	≤39	46 (25 F)	11.7	21.5 ± 1.6	73.9 ± 3.3	34.2 ± 3.8
Intermediate groups	<70 or >53	<54 or >39	296 (165 F)	75.51	21.6 ± 1.7	62.0 ± 8.7	46.3 ± 7.2
Low mindfulness and high anxiety^∗^	≤53	≥54	38 (17 F)	9.7	21.5 ± 1.6	45.0 ± 7.1	61.1 ± 5.3
Low mindfulness low anxiety	≤53	≤39	3 (3 F)	0.76	21.7 ± 0.6	47.0 ± 3.6	34.7 ± 3.8


*^∗^Experimental groups.*

Initially a ±1 SD criteria was used to place the subjects in two experimental groups, but this did not result in a sufficient number of subjects to reach an acceptable level of statistical power (with a desired power of 80% or above). We therefore reduced the threshold to ±0.85 SD to include sufficient subjects in the experimental groups. In general, many statistical books recommend recruiting at least 25 subjects to reach a valid significance level and, in addition, we wanted to have both experimental groups matched in as many as dimensions possible, as recommended by Davidson and Kaszniak (2015). We also kept an equal number of females and males in both groups, primarily as this was not a factor of interest. Another limitation when assigning participants to experimental groups was that there were some participants from both groups those were unwilling to take part in the experiment, generally due to time constrains. Since we had three behavioral tasks under investigation in a counter-balanced order, meaning we had always six combinations of orders of tasks, we kept the number of subjects in each group as a multiple of 6, resulting in 30 participants per group. Thus, there was a total of 60 participants (30 HMLA and 30 LMHA individuals) who completed the entire experimental procedure. Although no statistically valid comparisons could be made for the HMHA and LMLA groups, it may have been beneficial to consider data from these groups to some degree. However, this was not possible as all three participants in the LMLA group declined to participate.

The final experimental groups were 30 individuals in the HMLA group (15 females; mean age = 21.13 years, *SD* = 1.28 years; MAAS mean score = 74.7, *SD* = 3.1; STAI-T mean score = 33.8, *SD* = 3.6) and 30 participants in the LMHA group (15 females; mean age = 20.76 years, *SD* = 1.92 years, MAAS mean score = 44.7, *SD* = 7.4; STAI-T mean score = 60.4, *SD* = 5.2). All the participants had normal vision or corrected-to normal vision. The data of one outlier participant was excluded during analysis (the HMLA group) because his/her WMC was more than 2 SD from the group mean.

Each participant gave written informed consent in accordance with the Declaration of Helsinki before participating in the experiment. All the experimental procedures were approved by the Institutional Review Board of National Taiwan University, Taipei, Taiwan.

### Task Procedure

The subjects completed a battery of supplementary questionnaires (Chinese versions): FFMQ (Baer et al., 2006; Lee and Chao, 2012), a comprehensive measure of mindfulness; STAI-State (Spielberger et al., 1970; Shek, 1993), a measure of the level of anxiety at the given moment; BIS-11 (Patton et al., 1995; Huang et al., 2013), to quantify different kinds of impulsivity; and PSS-10 (Cohen et al., 1994; Wang et al., 2011), to measure the stress perceived by participants before they began the experiemnt. The FFMQ questionnaire measures five facets of mindfulness; acting with awareness, non-reaction to inner experience, non-judgment of inner experiences, observing inner experiences, and describe inner experiences. The BIS-11 questionnaire provides measures of three kinds of impulsivity; attentional, motor, and non-planning. These supplementary questionnaire responses were collected to investigate any additional indices which might be related to cognitive performance beyond the mindfulness/anxiety links that were the primary focus of the study.

All participants performed three cognitive tasks: an ANT (Fan et al., 2002), a CST (Stroop, 1935; Teper and Inzlicht, 2013), and a VWM (Luck and Vogel, 1997). Tasks were performed in a counter-balanced order across participants in both groups with a break of at least 2–5 min between the presentation of each. After completing the tasks, participants were either compensated with money or assigned course credits.

### Stimuli and Task Performance

All the stimuli were presented, and data were acquired using an IBM-compatible PC connected to a 23-inch LCD monitor and using Psychtoolbox-3 for MATLAB. The distance between the screen and participant was 60 cm.

#### Attentional Network Test (ANT)

The ANT paradigm was adapted from a study by Fan et al. (2007). A fixation cross was presented at the center of the screen for a variable period from 400 to 1600 ms. It was followed by appearance of a cue of one of the following types for 100 ms; a spatial-cue either above or below the fixation cross, a center-cue in place of the fixation cross, or no-cue at all. In the subsequent target presentation, an arrow horizontally flanked by two non-target arrows on each side was presented 500 ms after the onset of the cue. Depending on the direction of flankers relative to the target arrows, the target condition was either congruent (flankers in the same direction as the target) or incongruent (flankers in the opposite direction to the target). All the stimuli were black presented against a white background (luminance 200.5 cd m^-2^). A single arrow in the target stimuli spanned a visual angle of 0.55° and adjacent arrows were separated by 0.06° of visual angle. One entire target stimulus had a total width of 3.08° of visual angle (Fan et al., 2002). This task was performed under normal lighting in a closed room. Participants were instructed to press designated keys according to the direction of the central arrow, ‘v’ for left and ‘m’ for right. They were asked to respond as fast and as accurately as possible and the target remained on-screen until they made a response or for a maximum period of 1700 ms, whichever occurred first. After 24 practice trials, they began the formal task that was comprised of a total of 288 trials with an equal proportion of target conditions (144 each for congruent and incongruent trials) and an equal proportion of cue conditions (96 each of no-cue, central cue, and spatial cues).

#### Color Stroop Task (CST)

The CST paradigm was adapted from Teper and Inzlicht (2013) and used Chinese characters for different colors [red (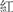), green (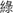), blue (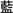), or yellow (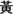)]. There were two types of targets. The first was a congruent target in which the color used to display the character matched its semantic meaning, and the second was an incongruent target, in which the word meaning and display color did not match. Each target stimulus was presented against a black background (2.6 cd m^-2^) at the center of screen within a rectangular area of 1.43° × 1.62° of visual angle. Participants had to identify the color used to display the characters and ignore the word meaning, responding by pressing the corresponding keys (‘d,’ ‘f,’ ‘k,’ and ‘l’) on a keyboard which had keys labeled with the colors. A single trial consisted of appearance of a fixation cross for 500 ms followed by the target character displayed for 200 ms. The participants were asked to respond as fast and as accurately as possible. There was a maximum 1000 ms duration for response collection with a blank screen that remained until they either pressed any key or until the time limit was reached. There was also a 1000 ms interval between trials and a total of 432 trials with a 2:1 ratio of congruent and incongruent conditions, which were pseudo-randomly distributed in such way that there were no more than three trials of an identical type in a row. Participants were given 72 practice trials before they began the formal experiment. The CST experiment was conducted in a closed dark room.

#### Visual Working Memory Task (VWM)

To assess the VWM capacity of both groups, a frequently used change detection paradigm (Luck and Vogel, 1997) was adapted and employed. Every trial began with a central fixation cross that was presented for 1000 ms, followed by a memory array comprised of 2, 4, or 8 colored squares presented in a random order and from a pool of highly discriminable colors (red, blue, violet, green, yellow, black, and white) and displayed for 100 ms. There was a blank display for a retention interval of 800 ms, succeeded by a test array with the same number of colored squares as in the memory array. Each of the colored squares in the stimulus arrays was of size 0.65° × 0.65° degrees and they were distributed within a 9.8° × 7.3° rectangular region. The distance between any two adjacent squares from center to center was at least 2°. There was a total of 288 trials with half of the trials with a change and half with no change. All three set sizes were presented equally frequently (96 trials for each set sizes) and for all 144 change trials there was an equal probability of change occurring in the left or right visual field. The participants had to detect and respond indicating if they thought the memory and test arrays were different or identical by pressing the ‘1’ or ‘2’ key, respectively. Before the formal experiment, each participant received 36 practice trials. In this paradigm directional cues were absent, so subjects were not given any visual field specific instruction about where to attend. All the stimuli were presented against a gray background (40.5 cd m^-2^) and the task was performed in a dimly lit closed room.

### Data Analysis

The two groups (HMLA and LMHA) and sexes (male and female) served as independent variables for the entire study. For analysis of variance (ANOVA), although it was not a factor of general interest for the analysis (and the number of participants of each gender was consequently lower than if this were to be investigated meaningfully) sex was included to check invariance. All the variables in each experiment were tested for normality using the Shapiro–Wilk test. Given the nature of tasks, we observed the accuracy rate in ANT and CST showed a high significance for this test, i.e., the data were non-normally distributed. We therefore employed non-parametric statistics (MWU) for the analysis of accuracy rates in these two tasks. For the VWM task, and in line with the recommendation of Rouder et al. (2011), to analyze WMC and other dependent variables, we dropped the analysis of the small set size condition (set size 2). To perform both parametric and non-parametric tests as appropriate, we used in-built syntaxes in SPSS software (PASW Statistics 18.0.0). In all statistical analyses, corrections for multiple comparisons employed Holm–Bonferroni corrections (Holm, 1979; Gaetano, 2018) as appropriate. All the effect sizes (Cohen’s *d*) (Cohen, 1988) and power analysis (1- β) were measured using version 3.0.10 of the G^∗^Power software (Faul et al., 2007). The effect sizes for the MWU are reported as Cohen’s *r* (Mayers, 2013).

#### Variables Analyzed for the Attentional Network Test

The efficiency of the attentional network was measured using the reaction time (RT) subtraction method (alerting score = no-cue RT – center-cue RT; orienting score = center-cue RT – spatial-cue RT; conflict score = incongruent target RT – congruent target RT) (Fan et al., 2007). The two groups were compared using independent *t*-tests on the above three network scores. For ANOVA, original RT measures (within subject factors), not the differences in RT measures (as described above) were employed according to previous studies (Fan et al., 2002; Jo et al., 2016). The accuracy rates under different cue and target conditions were analyzed by MWU. The primary behavioral measures of the ANT were alerting, orienting and conflict scores.

#### Variables Analyzed for the Color Stroop Task

The Stroop effect was measured by subtracting reaction times for the congruent target condition from that of the incongruent target conditions (within subject factors) (Stroop, 1935). The two groups were compared using independent *t*-tests on the Stroop effect. For ANOVA original RT measures, not the differences in RT measures (as described above) were employed according to previous studies (Fan et al., 2002; Jo et al., 2016). The accuracy rates under different target conditions were analyzed by MWU. The primary behavioral measures of the CST was the Stroop effect.

#### Variables Analyzed for the Visual Working Memory Task

The dependent variables for the VWM task were accuracy rate, d prime, and Kp for each set size of the task. Since RT measures for VWM task were not of critical importance, they were not analyzed or reported. D-prime values were calculated using the formula *d*′ = z(Hit rate) – z(False Alarm rate). WMC was measured using Kp (Pashler, 1988), appropriate for a whole-display tasks and employing the formula: Kp = Set Size ^∗^ [(Hit rate – False Alarm rate)/(1- False Alarm rate)] (Rouder et al., 2011). All the variables (accuracy rate and Kp values) except d-prime were found to be normally distributed for set sizes 4 and 8 (within subject factors). D-prime values were Box Cox power transformed (Box and Cox, 1964) using R Studio (version 1.0.136) such that the transformed values were reasonably normally distributed. The two groups were compared using independent *t*-tests on accuracy rate (index of general attention), d- prime values (index of sensitivity), and Kp values (index of WMC). The primary behavioral measures for the VWM task were Kp-4 and Kp-8 values, indexing WMC for set size 4 and set size 8.

## Results

### Attentional Network Task (ANT)

A mixed four-way ANOVA was conducted on RTs for cue and target types, where group (HMLA versus LMHA) and sex (male or female) served as between subject factors, while cue types (no-cue, center-cue, and spatial-cue) and target types (congruent versus incongruent) served as within subject factors. This showed a significant main effect of cue [*F* = 533.218, *p* < 0.001] and of target [*F* = 460.315, *p* < 0.0001], but not of group type [*F* = 0.021, *p* = 0.885] and sex [*F* = 0.664, *p* = 0.419]. There was a significant interaction between cue and target [*F* = 20.568, *p* < 0.001], but no interaction was observed between cue and group [*F* = 0.410, *p* = 0.665], between target and group [*F* = 0.027, *p* = 0.870], between sex and group [*F* = 1.088, *p* = 0.302], between cue and sex [*F* = 0.007, *p* = 0.993] or between target and sex [*F* = 1.897, *p* = 0.174]. There was no interaction for cue, target, and group [*F* = 0.016, *p* = 0.984], nor for cue, target, and sex [*F* = 1.320, *p* = 0.271], target, sex, and group [*F* = 0.440, *p* = 0.510] nor for cue, sex, and group [*F* = 1.109, *p* = 0.334]. There was no four-way interaction for target, cue, sex, and group [*F* = 0.378, *p* = 0.686]. The effect sizes (*d*) and statistical power (*1-*β) for the RT measures for all the cue and target conditions were smaller than 0.1 and 0.07 respectively. The independent *t*-test results on three attentional networks did not show any group differences (**Table 2**).

**Table 2:** Summary of attentional network efficiency scores (mean ± SEM).

	Alerting score (ms)	Orienting score (ms)	Conflict score (ms)
HMLA group	46.07 ± 3.8	60.94 ± 4.36	86.54 ± 6.09
LMHA group	41.88 ± 4.63	67.05 ± 4.14	84.50 ± 5.24
*t*-test ^∗^*p*-value	0.976	0.942	0.976


*^∗^*p*-Values reported here are after Holm–Bonferroni corrections.*

The Independent MWU test was performed on accuracy rate for each cue and target type combination to compare the accuracy rate between the two groups. This did not show any significant difference between the two groups for any of the cue-target combinations (**Table 3**).

**Table 3:** Summary of median accuracy rate (%) on the ANT across cue and target conditions [median (lower CI – upper CI)].

	No cue congruent	No cue incongruent	Center cue congruent	Center cue incongruent	Spatial cue congruent	Spatial cue incongruent
HMLA group	100 (100–100)	97.92 (97.92–97.92)	100 (100–100)	97.92 (95.83–97.92)	100 (100–100)	100 (100–100)
LMHA group	100 (100–100)	97.83 (94.79–97.92)	100 (100–100)	95.83 (93.75–97.92)	100 (100–100)	97.92 (97.83–100)
# MWU-test ^∗^*p*-value	< = 1.000	< = 1.000	< = 1.000	< = 1.000	< = 1.000	< = 1.000


**^∗^p*-Values reported here are after Holm–Bonferroni corrections. ^#^Non-parametric test.*

### Color Stroop Task (CST)

A mixed three-way ANOVA was conducted on RTs where group (HMLA versus LMHA) and sex (male or female) served as between subject factors, while target type (congruent versus incongruent) was a within subject factor. ANOVA of RTs showed a main effect of the target [*F* = 229.340, *p* < 0.0001], but neither of group [*F* = 0.400, *p* = 0.530] nor of sex [*F* = 0.072, *p* = 0.789]. There was also no significant interaction observed between target and group [*F* = 0.504, *p* = 0.481], between sex and group [*F* = 0.381, *p* = 0.540], or for target and sex [*F* = 0.270, *p* = 0.606]. There was no three-way interaction for target, sex and group [*F* = 0.383, *p* = 0.538]. The effect sizes (*d*) and statistical powers (*1-*β) on RT measures for the congruent target were 0.25 and 0.16 respectively, while effect sizes (*d*) and statistical powers (*1-*β) for the incongruent target were 0.10 and 0.07 respectively. An independent *t*-test on Stroop effect showed no significant difference between the two groups (HMLA group 98.55 ± 9.15 ms, LMHA group 89.51 ± 8.18, *t* = 0.738, *p* = 0.463).

**FIGURE 1:**
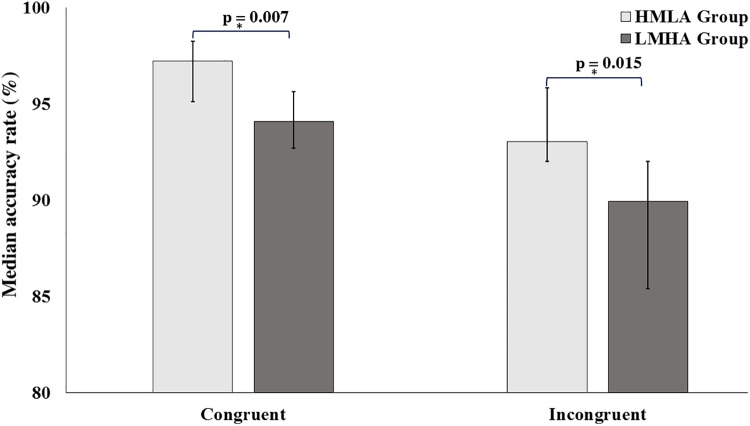
Median Accuracy rate (%) on the CST in congruent and incongruent conditions. Error bars represent bias corrected 95% confidence intervals. The MWU showed that the HMLA group were better in accuracy in both the congruent (*r* = 0.349, *p* = 0.014) and incongruent (*r* = 0.316, *p* = 0.015) conditions. ^∗^*p*-Values reported here are after Holm–Bonferroni corrections.

The accuracy rate analyzed using the MWU test showed that the HMLA group performed the task more accurately than the LMHA group for both the congruent and incongruent conditions (**Figure 1**).

### Visual Working Memory Task

A mixed three-way ANOVA, where group (HMLA versus LMHA) and sex (male or female) served as between subject factors and set sizes (4 and 8) served as a within subject factor, was performed on accuracy rate, Box Cox transformed d prime values (*lambda_size_*_4_ = 0.145, *lambda*_*size*__8_ = 0.926) and Kp values.

**FIGURE 2:**
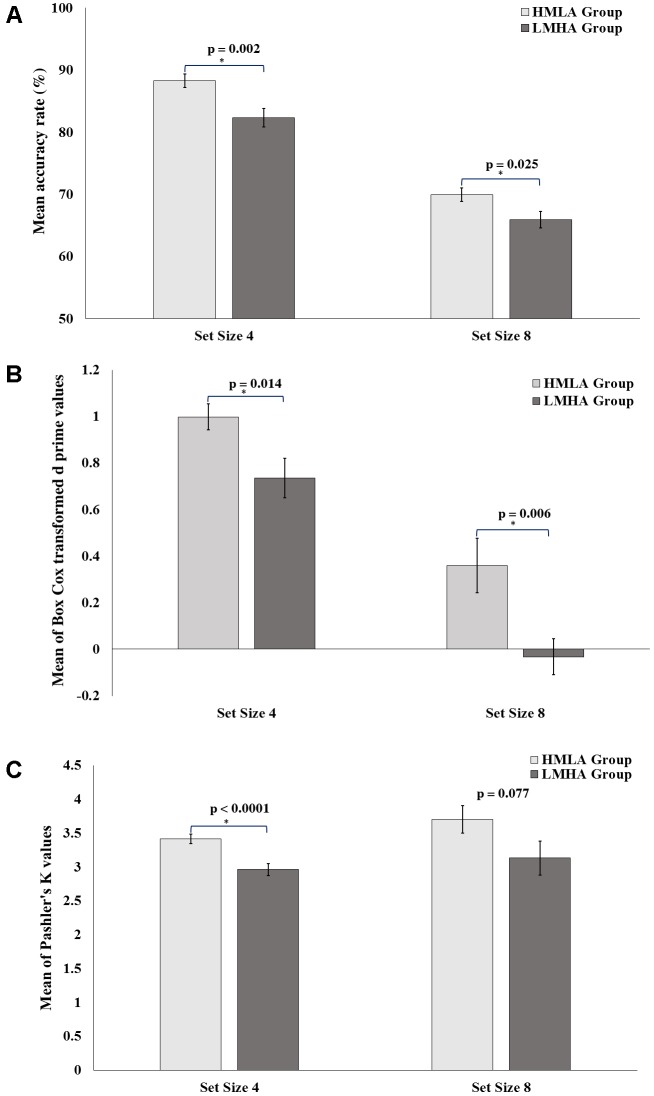
Results from the VWM change detection task and comparison in performance between the HMLA and LMHA groups. **(A)** Mean accuracy rate (%) for set size 4 and set size 8. **(B)** Mean of Box Cox power transformed d prime values for set size 4 and set size 8. **(C)** Mean of Kp for set size 4 and set size 8. Error bars represent standard errors of the mean. ^∗^*p*-Values reported here are after Holm–Bonferroni corrections.

The repeated measure ANOVA of accuracy revealed a significant main effect of set size [*F* = 455.693, *p* < 0.0001] as well as of group type [*F* = 10.414, *p* = 0.002], but no main effect of sex [*F* = 0.246, *p* = 0.622]. However, there was no significant interaction between the set size and group [*F* = 1.430, *p* = 0.237], between set size and sex [*F* = 3.389, *p* = 0.071], or between sex and group [*F* = 3.592, *p* = 0.063]. There was no three-way interaction among set size, sex, and group [*F* = 1.053, *p* = 0.309]. The independent *t*-tests showed significant difference between the two groups on both set sizes (**Figure 2A**). The effect sizes (*d*) for the accuracy measures under set size 4 and set size 8 target conditions were 0.86 and 0.61 respectively, while the statistical power (*1-*β) under set size 4 and set size 8 target conditions were 0.90 and 0.63 respectively.

For the d prime analysis, ANOVA results showed a significant main effect of set size [*F* = 139.494, *p* < 0.0001] and also of group type [*F* = 10.519, *p* = 0.002], but no main effect of sex [*F* = 0.386, *p* = 0.537]. There were two-way interactions observed between sex and group [*F* = 4.999, *p* = 0.029] as well as between sex and set size [*F* = 6.099, *p* = 0.017], but no significant interaction was observed between set size and group [*F* = 1.332, *p* = 0.253]. There was no significant three-way interaction between set size, sex, and group [*F* = 0.468, *p* = 0.497]. Further, *post hoc t*-tests on the interaction between sex and group revealed that only male participants of the HMLA group showed significantly higher transformed d-prime values than those in the LMHA group for set size 4 (HMLA group: 1.053 ± 0.062; LMHA group: 0.601 ± 0.115; *t* = 3.449, *p* = 0.002) and for set size 8 (HMLA group: 0.609 ± 0.199; LMHA group: -0.063 ± 0.096; *t* = 3.109, *p* = 0.004). There was no such difference for females either for set size 4 (HMLA group: 0.949 ± 0.093; LMHA group: 0.871 ± 0.119; *t* = 0.516, *p* = 0.610) or for set size 8 (HMLA group: 0.129 ± 0.102; LMHA group: -0.013 ± 0.122; *t* = 0.815, *p* = 0.442). Additionally, *post hoc* independent *t*-tests for the interaction between sex and set size did not indicate significant differences between males and females in transformed d-prime values on set size 4 (males: 0.819 ± 0.078; females: 0.909 ± 0.074; *t* = -0.839, *p* = 0.405) or on set size 8 (males: 0.261 ± 0.123; females: 0.063 ± 0.079; *t* = 1.363, *p* = 0.178). The independent *t*-tests for the transformed d-prime values showed significant difference between the two groups on both set sizes (**Figure 2B**). The effect sizes (*d*) for the transformed d-prime values under set size 4 and set size 8 target conditions were 1.13 and 2.87 respectively, while statistical power (*1-*β) under set size 4 and set size 8 target conditions were 0.99 and < = 1 respectively.

The ANOVA carried out on Kp values showed no main effect of set size [*F* = 2.717, *p* = 0.105] or sex [*F* = 0.833, *p* = 0.365], but a significant main effect of group type [*F* = 7.220, *p* = 0.010]. There were no two-way interactions between set size and group [*F* = 0.218, *p* = 0.643], between set size and sex [*F* = 2.624, *p* = 0.111] or between sex and group [*F* = 0.872, *p* = 0.355]. Moreover, there was no three-way interaction observed for set size, sex, and group [*F* = 0.017, *p* = 0.896]. The independent *t*-tests showed significant difference between the two groups on both set sizes (**Figure 2C**). The effect sizes (*d*) for the Kp values under set size 4 and set size 8 target conditions were 1.04 and 0.48 respectively, while statistical power (*1-*β) under the set size 4 and set size 8 target conditions were 0.98 and 0.43 respectively.

### Correlation Between Primary Behavioral Measures and Trait Mindfulness and Trait Anxiety

**FIGURE 3:**
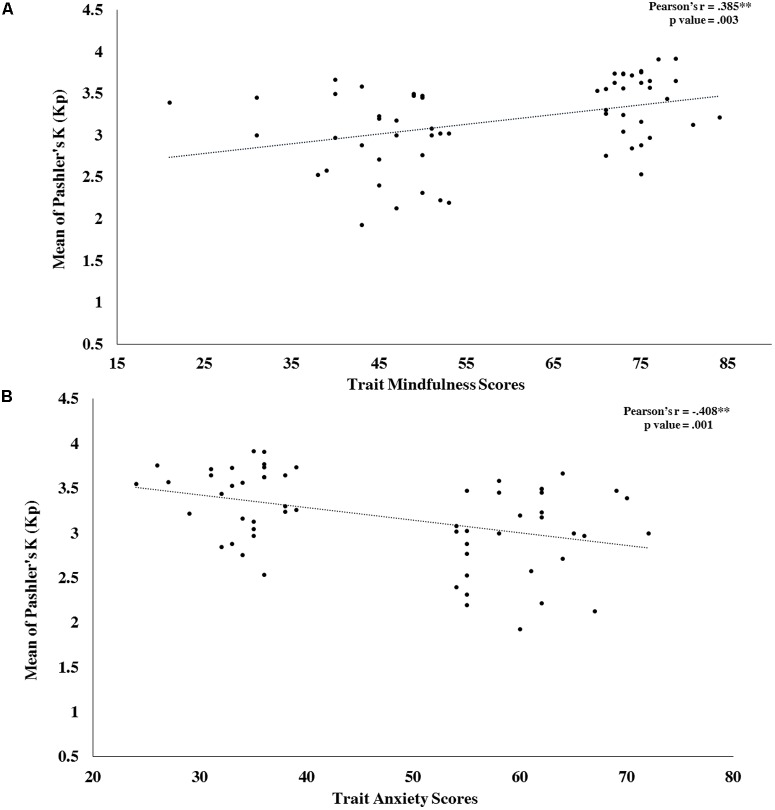
Only WMC under set 4 could be predicted by both mindfulness as well as anxiety scores. Therefore, two-dimensional plots of mindfulness and anxiety scores with corresponding Kp values for set size 4 are presented here. **(A)** Mindfulness Scores versus WMC for set size 4. **(B)** Anxiety Scores versus WMC for set size 4. ^∗^*p*-Values reported here are after Holm–Bonferroni corrections. ^∗∗^*p* < 0.01.

The participants of both groups (HMLA and LMHA) were pooled together in two categories based on their trait mindfulness and trait anxiety scores and investigated for the nature of the distribution of their primary behavioral measures (alerting, orienting and conflict scores for ANT, Stroop effect for CST and WMC for VWM) using Pearson’s correlation. It was observed that, except for Kp-4 values, neither mindfulness nor anxiety was correlated with the distribution of primary behavioral measures (**Table 4**). Further, a two-dimensional plot between mindfulness scores and Kp value for set size 4 showed a direct positive relationship (**Figure 3A**), while the plot between anxiety scores and Kp value for set size 4 showed an inverse negative relationship (**Figure 3B**).

**Table 4:** Correlations between primary behavioral measures, trait mindfulness, and trait anxiety independently.

	Alerting	Orienting	Conflict	Stroop effect	Kp-4	Kp-8
Mindfulness scores (Pearson’s *r*)	0.126	-0.051	0.008	0.093	0.385^∗^	0.211
*p*-Values	< = 1	< = 1	< = 1	< = 1	0.033	0.972
Trait anxiety scores (Pearson’s *r*)	0.002	0.037	-0.037	-0.177	-0.408^∗^	-0.258
*p*-Values	< = 1	<= 1	< = 1	< = 1	0.012	0.090


*Kp-4 and Kp-8 WMC under set sizes 4 and 8. None of the variables except Kp-4 value, that is, WMC under set size 4 could be predicted by both primary self-report measures independently. *^∗^p*-Values reported here are after Holm–Bonferroni corrections.*

### Supplementary Questionnaires

There was a total of 10 predictors (described in the procedures) estimated from supplementary self-report measures that were used as potential factors in an explanatory model that might describe variability in primary behavioral measures. The questionnaire scores: FFMQ (α = 0.896), STAI-state (α = 0.896), BIS-11 (α = 0.847), and PSS-10 (α = 0.919) were analyzed using multiple regression (stepwise) on primary behavioral measures of the three tasks (attentional networks for ANT, Stroop effect for CST and WMC for VWM) after pooling individuals (**Table 5**). As shown in the table, there were only a few independent variables that were related to the primary behavioral measures such as orienting, Stroop effect, and WMC under set size 4. The percentages of variance explained by these predictors on the behavioral measures were quite small (<20%).

**Table 5:** Regression models predicted by supplementary self-report measures on primary behavioral measures.

Dependent variable (predictor variables)	Model	*R*^2^	Adjacent *R*^2^	*F*-Value (ANOVA)	*p*-Value (ANOVA)	Gradient	*t*-Value	*p*-Value
Alerting	o							
Orienting (Perceived Stress Scale^a^, describe inner experiences^b^)	2	0.154	0.124	5.103	0.009^∗∗^	0.345^a^	2.715	0.009^∗∗^
						0.294^b^	2.315	0.024^∗^
Conflict	o							
Stroop effect (non-judgment to inner experiences^c^)	1	0.103	0.088	6.574	0.013^∗^	0.322	2.564	0.013^∗^
Kp-4 (act with awareness^d^)	1	0.196	0.182	13.866	<0.001^∗∗^	0.442	3.724	<0.001^∗∗^
Kp-8	o							


*(1) Model number 1 that explained maximum variance, (2) model number 2 that explained maximum variance, (o) no significant model could be predicted. ^*a*^Perceived Stress Scale, ^*b*^describe inner experiences, ^*c*^non-judgment to inner experiences, ^*d*^act with awareness. Kp-4 and Kp-8 WMC under set sizes 4 and 8. ^∗∗^*p* < 0.01; ^∗^*p* < 0.05.*

## Discussion

The current research investigated whether differences in trait mindfulness and trait anxiety would be reflected in differences in cognitive functions in a normal, non-meditating population. In the initial sample of 392 participants a strong negative correlation between trait mindfulness and trait anxiety in the sample pool was seen, consistent with prior reports (Coffey and Hartman, 2008; Desrosiers et al., 2013). To our knowledge, this is the first study that has examined how two inversely related traits, dispositional mindfulness, and trait anxiety may have bearings on executive functions. We observed that the HMLA group individuals performed better than the LMHA group on the CST and the VWM tasks in terms of task accuracy, sensitivity, and WMC, but did not differ in reaction time measures. Interestingly, the interaction between sex and group on the change detection task indicated that only male participants differing in their mindfulness and anxiety scores showed an advantage in sensitivity as indexed by transformed d-prime values, but no such pattern was observed among the female participants. However, males and females did not differ in sensitivity for set size 4 or for set size 8, as reflected by *post hoc t*-tests on the interaction between sex and set size. In addition, there was a trend toward higher accuracy in the incongruent conditions of the ANT for the HMLA group than for the LMHA group.

The current study did not observe any sex differences in the rating of anxiety for the overall sample pool or within the experimental groups. This was quite inconsistent with many previous studies that frequently report females showing higher levels of anxiety (e.g., McLean et al., 2011; Donner and Lowry, 2013). A possibility of sampling error could be one reason for this observation (Assael and Keon, 1982; Dillman and Bowker, 2001). However, this inconsistency in self-report could also be due to social-cultural differences. A recent study on depression in Taiwan observed no sex differences in depressive symptoms (Chang et al., 2013). There was also a recent mediational study (Zalta and Chambless, 2012) in which, after controlling for stress and social desirability, no direct significant link between gender and anxiety was observed. Hence, invariance observed between sexes in the current study on self-report measures as well as on behavioral variables is plausible.

The supplementary self-report measures showed some of the behavioral measures such as orienting, Stroop effect and Kp value under set size 4 were indeed were moderated across the groups. However, the percentage of variance explained by the self-report measures was quite low (<20%), making it difficult to make any meaningful interpretation from this observation. An additional limitation while performing regression analysis was faced pertaining to the required sample size, with the minimum required sample size defined by a formula (Tabachnick and Fidell, 2007) N ≥ 50 + 8m (where m = number of predictors and N = sample size) (Mayers, 2013) that the current study could not fulfill (required sample size ≥ 130).

For the ANT, we did not observe any significant differences between the two groups in the efficiency of any of the three attentional subsystems, consistent with a previous cross-sectional study on meditation (Jo et al., 2016). Although this previous study found that meditators showed an overall higher accuracy rate than control group, the current study did not find any significant differences between the HMLA and LMHA groups. There are two independent studies on dispositional mindfulness (Di Francesco et al., 2017) and trait anxiety (Pacheco-Unguetti et al., 2010) that have shown that these two factors modulate the efficiency of attentional networks. However, the current study is quite different in design from these two studies and did not see such an effect. This difference could also be due to the use of a modified version of the ANT-I (Callejas et al., 2004) in these two studies, which was done to study interactions between three networks. In ANT-I, a short, high-frequency auditory tone is presented as an alerting stimulus, whereas in the current study all cues were only presented visually. Although both studies had a comparable number of participants, the measures employed to estimate dispositional mindfulness could be another factor contributing to a difference in results. The current study employed a single questionnaire developed by Brown and Ryan (2003), while Di Francesco et al. (2017) used a FFMQ (Baer et al., 2006). Additionally, their analysis involved continuous correlation between behavioral performance and the facets of mindfulness scores, whereas in the current study two relatively extreme groups of individuals were selected and compared in terms of behavioral performance.

In the current study, for the CST our findings were in line with a previous cross-sectional study on meditation (Teper and Inzlicht, 2013), in that we did not observe a group difference in the Stroop effect, although there was a pattern that the HMLA group were more accurate than the LMHA group. This observation also partially echoed the findings of Moore and Malinowski (2009), where they observed that a high mindfulness score was correlated with a lower error rate and higher accuracy in both meditator and control groups. They further suggested that there is a relationship between dispositional mindfulness and cognitive inhibition. Several previous studies have employed emotional versions of the Stroop task to investigate the effect of anxiety and showed that anxious subjects had a greater Stroop effect on reaction time measures (de Ruiter and Brosschot, 1994; Becker et al., 2001). The current study, which did not require processing of affective information, did not observe such an effect. In terms of the general reaction time measure, the Stroop effect is the primary dependent variable which provides the measure of cognitive conflict (Miyake et al., 2000) and in which no differences were seen in the current study. However, the higher accuracy for the HMLA group than for the LMHA group indicates better overall general attention in HMLA group reflecting better motor control (Heitz and Schall, 2012; Tarrasch et al., 2017).

Most of the previous WMC studies on meditation have employed either an operation span task (Jha et al., 2010; Mrazek et al., 2013) or a n-back task (Zeidan et al., 2010), primarily focusing on the verbal storage system. To our knowledge the current study is the first to employ a change detection paradigm (Luck and Vogel, 1997), providing a measure of visuospatial WMC in relation to mindfulness. However, one recent study has explored WMC using a change detection paradigm in relation to trait anxiety (Moriya and Sugiura, 2012), but their observations were quite contrary to our current findings. They found that people with higher social anxiety had a higher WMC than those with lower anxiety, while the present research found otherwise. This difference in findings may be due to the way WMC was estimated in each study. In the present study WMC was estimated for set size 4 and set size 8 independently for two groups, in comparison with the previous study of WMC which used higher set sizes of 8 and 12 and which were averaged and compared for continuous correlation with social anxiety scores of participants. The literature suggests that human working memory has an optimum capacity of around 4 to 5 items (see the review of Cowan, 2010). Thus set size 4 in the current study could serve as suitable index to discern differences in capacity of two different populations at around the expected capacity limit. The set size 8 could show the differences in capacity when it reaches at saturation point, near, at, or above the near upper limit of WMC (Todd and Marois, 2004; Luck and Vogel, 2013). Therefore, the data here suggests that the HMLA group indeed had better WMC than the LMHA group even when demands on WM resources were higher. A higher WMC for both sizes 4 and 8 in the HMLA group indicates better ability in retaining visuo-spatial information for these individuals.

Another reason for difference in the current study from that of the Moriya and Sugiura (2012) could be due to the difference in the measures of anxiety used in the two studies, with Moriya and Sugiura (2012) measuring social anxiety, whereas the current study used a more general measure of anxiety, STAI-Trait. In the current study, a direct positive correlation between mindfulness scores and WMC as well as an inverse negative correlation between anxiety scores and WMC may further indicate that WMC is sensitive to personality traits.

The current study investigated two cross-sectional groups without any previous meditation experience. By recruiting participants without any prior meditation experience, it was possible that some of the difficulties associated with matching controls that can occur in meditation-related studies were reduced, although it should be noted that meditators and high-mindfulness individuals are by no means equivalent. By selecting subjects from a large sample pool based on a double selection criterion, we also reduced the likelihood of inclusion of individuals with overlapping traits due to reasons less linked to the mindfulness/anxiety association, so better characterizing effects presumably due to a more specific, emotion regulation pathway difference.

One major limitation of the current study is that the other two extreme groups from the sample population, HMHA and LMLA groups were not included. Unless the four groups are contrasted (LMHA, HMLA, LMLA, and HMHA), it is theoretically not possible to determine whether any of the effects detected are due to either a trait alone or in combination. Hence, it would be useful to investigate these groups in the future, although the numbers in the present study indicate that a potentially very large number of individuals would have to be screened for this to be plausible. Another important limitation of this research is that it is correlational and therefore cannot propose causative explanations and that the effects reported here may be a result of other factors. Additionally, although it was discussed how trait mindfulness and trait anxiety could be linked via emotion regulation strategy, the current study did not employ any objective measure that could account for differences in emotion regulation strategies between the two groups. Moreover, the current study did not have enough each sex in the experimental groups to observe any main effect of sex, or to rule out that there might be such an effect. Future research should ideally employ a larger sample size and explore the underlying fundamental mechanisms of emotion regulation and how any changes in such regulation strategies relate to levels of mindfulness and anxiety, and consequently to executive functions.

## Conclusion

This study examined how mindfulness and anxiety together may affect executive control using the ANT, the CST, and a change detection task for two groups: one of HMLA individuals and one of LMHA individuals. Results showed the HMLA group were more accurate than the LMHA group on both congruent and incongruent conditions in the CST and also on the change detection task. Moreover, the HMLA groups were found to be more sensitive and had a higher WMC. The pattern of performance, and differences between the two groups, are in line with the suggested inverse link between mindfulness and anxiety having specific effects on cognitive measures. It is possible that the emotion regulation mechanism may be a common link between the two traits and future studies might beneficially explore how emotion regulation strategy may be associated with different cognitive functions. Also of benefit would be investigation of electrophysiological measure and adaptability associated with these two linked traits, potentially further revealing the underlying neural mechanism behind the behavioral differences observed here.

## Author Contributions

SJ: designed the study, collected and analyzed the data, and was involved in preparation of the manuscript. S-YT: data acquisition and revision of the manuscript. C-HJ, W-KL, and NM: study design, data interpretation, and revision of the manuscript. All have approved the final version of the manuscript and are accountable for the work described.

## Conflict of Interest Statement

The authors declare that the research was conducted in the absence of any commercial or financial relationships that could be construed as a potential conflict of interest.

## Abbreviations

ANT: attentional network test
BIS-11: Barratt’s Impulsivity Scale
CI: confidence interval
CST: color Stroop task
FFMQ: Five Facets of Mindfulness Questionnaire
HMLA: high mindfulness and low anxiety group
Kp: Pashler’s *K*-value
LMHA: low mindfulness and high anxiety group
MAAS: Mindful Attention Awareness Scale
MWU: Mann–Whitney *U-*test
PFC: prefrontal cortex
PSS-10: Perceived Stress Scale
r: Effect size for MWU test
SD: standard deviation
SEM: standard error about mean
STAI: State Trait Anxiety Inventory
VWM: visual working memory
WMC: working memory capacity

## References

[B1] Aiken L. S. West S. G. Reno R. R. (1991). *Multiple Regression: Testing and Interpreting Interactions.* Thousand Oaks, CA: Sage.

[B2] Assael H. Keon J. (1982). Nonsampling vs. sampling errors in survey research. *J. Mark.* 46 114–123. 10.2307/3203346

[B3] Baer R. A. Smith G. T. Hopkins J. Krietemeyer J. Toney L. (2006). Using self-report assessment methods to explore facets of mindfulness. *Assessment* 13 27–45. 10.1177/1073191105283504 16443717

[B4] Baijal S. Jha A. P. Kiyonaga A. Singh R. Srinivasan N. (2011). The influence of concentrative meditation training on the development of attention networks during early adolescence. *Front. Psychol.* 2:153. 10.3389/fpsyg.2011.00153 21808627 PMC3137946

[B5] Barlow D. H. (2004). *Anxiety and its Disorders: The Nature and Treatment of Anxiety and Panic.* New York, NY: Guilford press.

[B6] Becker E. S. Rinck M. Margraf J. Roth W. T. (2001). The emotional Stroop effect in anxiety disorders: general emotional or disorder specificity? *J. Anxiety Disord.* 15 147–159. 10.1016/S0887-6185(01)00055-X 11442135

[B7] Berggren N. Derakshan N. (2013). Attentional control deficits in trait anxiety: why you see them and why you don’t. *Biol. Psychol.* 92 440–446. 10.1016/j.biopsycho.2012.03.007 22465045

[B8] Berryhill M. E. Olson I. R. (2008). Is the posterior parietal lobe involved in working memory retrieval? Evidence from patients with bilateral parietal lobe damage. *Neuropsychologia* 46 1767–1774. 10.1016/j.neuropsychologia.2008.01.009 18439630 PMC2494709

[B9] Bishop S. J. (2009). Trait anxiety and impoverished prefrontal control of attention. *Nat. Neurosci.* 12 92–98. 10.1038/nn.2242 19079249

[B10] Bishop S. R. Lau M. Shapiro S. Carlson L. Anderson N. D. Carmody J. (2004). Mindfulness: a proposed operational definition. *Clin. Psychol. Sci. Pract.* 11 230–241. 10.1093/clipsy.bph077

[B11] Box G. E. P. Cox D. R. (1964). An analysis of transformations. *J. R. Stat. Soc. Ser. B* 26 211–252.

[B12] Brown K. W. Goodman R. J. Inzlicht M. (2013). Dispositional mindfulness and the attenuation of neural responses to emotional stimuli. *Soc. Cogn. Affect. Neurosci.* 8 93–99. 10.1093/scan/nss004 22253259 PMC3541486

[B13] Brown K. W. Ryan R. M. (2003). The benefits of being present: mindfulness and its role in psychological well-being. *J. Pers. Soc. Psychol.* 84 822–848. 10.1037/0022-3514.84.4.822 12703651

[B14] Callejas A. Lupiáñez J. Tudela P. (2004). The three attentional networks: on their independence and interactions. *Brain Cogn.* 54 225–227. 10.1016/j.bandc.2004.02.012 15050779

[B15] Chang J. H. Huang C.-L. Lin Y.-C. (2015). Mindfulness, basic psychological needs fulfillment, and well-being. *J. Happiness Stud.* 16 1149–1162. 10.1016/j.cpr.2010.03.001 21151705 PMC2998793

[B16] Chang Y. Li T. Teng H. Y. Berki A. Chen L. H. (2013). Living with gratitude: spouse’s gratitude on one’s depression. *J. Happiness Stud.* 14 1431–1442. 10.1007/s10902-012-9389-4

[B17] Cisler J. M. Olatunji B. O. (2012). Emotion regulation and anxiety disorders. *Curr. Psychiatry Rep.* 14 182–187. 10.1007/s11920-012-0262-2 22392595 PMC3596813

[B18] Coffey K. A. Hartman M. (2008). Mechanisms of action in the inverse relationship between mindfulness and psychological distress. *Complement. Health Pract. Rev.* 13 79–91. 10.1177/1533210108316307

[B19] Cohen J. (1988). *Statistical Power Analysis for the Behavioral Sciences*, 2nd Edn Hillsdale, NJ: Lawrence Erlbaum.

[B20] Cohen S. Kamarck T. Mermelstein R. (1994). “Perceived stress scale. measuring stress,” in *A Guide for Health and Social Scientists*, eds Cohen S. Kessler R. C. Gordon L. U. (New York, NY: Oxford University Press).

[B21] Cosme D. Wiens S. (2015). Self-Reported trait mindfulness and affective reactivity: a motivational approach using multiple psychophysiological measures. *PLoS One* 10:e0119466. 10.1371/journal.pone.0119466 25749431 PMC4352075

[B22] Cowan N. (2010). The magical mystery four: how is working memory capacity limited, and why. *Curr. Dir. Psychol. Sci.* 19 51–57. 10.1177/0963721409359277 20445769 PMC2864034

[B23] Creswell J. D. Way B. M. Eisenberger N. Lieberman M. D. (2007). Neural correlates of dispositional mindfulness during affect labeling. *Psychosom. Med.* 69 560–565. 10.1097/PSY.0b013e3180f6171f 17634566

[B24] Darke S. (1988). Anxiety and working memory capacity. *Cogn. Emot.* 2 145–154. 10.1080/02699938808408071

[B25] Davidson R. J. (2002). Anxiety and affective style: role of prefrontal cortex and amygdala. *Biol. Psychiatry* 51 68–80. 10.1016/S0006-3223(01)01328-2 11801232

[B26] Davidson R. J. Kaszniak A. W. (2015). Conceptual and methodological issues in research on mindfulness and meditation. *Am. Psychol.* 70 581–592. 10.1037/a0039512 26436310 PMC4627495

[B27] Davis M. (1992). The role of the amygdala in fear and anxiety. *Annu. Rev. Neurosci.* 15 353–375. 10.1146/annurev.ne.15.030192.002033 1575447

[B28] de Ruiter C. Brosschot J. F. (1994). The emotional Stroop interference effect in anxiety: attentional bias or cognitive avoidance. *Behav. Res. Ther.* 32 315–319. 10.1016/0005-7967(94)90128-7 8192630

[B29] Desrosiers A. Vine V. Klemanski D. H. Nolen-Hoeksema S. (2013). Mindfulness and emotion regulation in depression and anxiety: common and distinct mechanisms of action. *Depress. Anxiety* 30 654–661. 10.1002/da.22124 23592556 PMC4012253

[B30] Di Francesco S. A. Simione L. Lopez-Ramon M. F. Belardinelli M. O. Lupianez J. Raffone A. (2017). Dispositional mindfulness facets predict the efficiency of attentional networks. *Mindfulness* 8 101–109. 10.1007/s12671-016-0634-5

[B31] Díaz-Morán S. Palència M. Mont-Cardona C. Cañete T. Blázquez G. Martínez-Membrives E. (2013). Gene expression in amygdala as a function of differential trait anxiety levels in genetically heterogeneous NIH-HS rats. *Behav. Brain Res.* 252 422–431. 10.1016/j.bbr.2013.05.066 23777796

[B32] Dillman D. A. Bowker D. (2001). The web questionnaire challenge to survey methodologists. *Online Soc. Sci.* 53–71.

[B33] Dobson P. (2000). An investigation into the relationship between neuroticism, extraversion and cognitive test performance in selection. *Int. J. Select. Assess.* 8 99–109. 10.1111/1468-2389.00140

[B34] Donner N. C. Lowry C. A. (2013). Sex differences in anxiety and emotional behavior. *Pflugers Arch.* 465 601–626. 10.1007/s00424-013-1271-7 23588380 PMC3805826

[B35] Etkin A. Klemenhagen K. C. Dudman J. T. Rogan M. T. Hen R. Kandel E. R. (2004). Individual differences in trait anxiety predict the response of the basolateral amygdala to unconsciously processed fearful faces. *Neuron* 44 1043–1055. 10.1016/j.neuron.2004.12.006 15603746

[B36] Eysenck M. W. Calvo M. G. (1992). Anxiety and performance: the processing efficiency theory. *Cogn. Emot.* 6 409–434. 10.1080/02699939208409696

[B37] Eysenck M. W. Derakshan N. Santos R. Calvo M. G. (2007). Anxiety and cognitive performance: attentional control theory. *Emotion* 7 336–353. 10.1037/1528-3542.7.2.336 17516812

[B38] Fan J. Byrne J. Worden M. S. Guise K. G. McCandliss B. D. Fossella J. (2007). The relation of brain oscillations to attentional networks. *J. Neurosci.* 27 6197–6206. 10.1523/JNEUROSCI.1833-07.2007 17553991 PMC6672149

[B39] Fan J. McCandliss B. D. Sommer T. Raz A. Posner M. I. (2002). Testing the efficiency and independence of attentional networks. *J. Cogn. Neurosci.* 14 340–347. 10.1162/089892902317361886 11970796

[B40] Faul F. Erdfelder E. Lang A. G. Buchner A. (2007). GPower 3: a flexible statistical power analysis program for the social, behavioral, and biomedical sciences. *Behav. Res. Methods* 39 175–191. 10.3758/BF03193146 17695343

[B41] Frewen P. A. Dozois D. J. Neufeld R. W. Lane R. D. Densmore M. Stevens T. K. (2010). Individual differences in trait mindfulness predict dorsomedial prefrontal and amygdala response during emotional imagery: an fMRI study. *Pers. Individ. Diff.* 49 479–484. 10.1016/j.paid.2010.05.008

[B42] Gaetano J. (2018). *Holm-Bonferroni Sequential Correction: An Excel Calculator (v. 1.3) Microsoft Excel Workbook.* Available at: https://www.researchgate.net/publication/322569220_Holm-Bonferroni_sequential_correction_An_Excel_calculator_13

[B43] Gallant S. N. (2016). Mindfulness meditation practice and executive functioning: breaking down the benefit. *Conscious. Cogn.* 40 116–130. 10.1016/j.concog.2016.01.005 26784917

[B44] Gold A. L. Morey R. A. McCarthy G. (2015). Amygdala–prefrontal cortex functional connectivity during threat-induced anxiety and goal distraction. *Biol. Psychiatry* 77 394–403. 10.1016/j.biopsych.2014.03.030 24882566 PMC4349396

[B45] Greeson J. Brantley J. (2009). “Mindfulness and anxiety disorders: developing a wise relationship with the inner experience of fear,” in *Clinical Handbook of Mindfulness*, ed. Fabrizio D. (Berlin: Springer Science & Business Media).

[B46] Heitz R. P. Schall J. D. (2012). Neural Mechanisms of speed-accuracy tradeoff. *Neuron* 76 616–628. 10.1016/j.neuron.2012.08.030 23141072 PMC3576837

[B47] Hofmann S. G. Sawyer A. T. Witt A. A. Oh D. (2010). The effect of mindfulness-based therapy on anxiety and depression: a meta-analytic review. *J. Consult. Clin. Psychol.* 78 169–183. 10.1037/a0018555 20350028 PMC2848393

[B48] Holm S. (1979). A simple sequentially rejective multiple test procedure. *Scand. J. Stat.* 6 65–70.

[B49] Huang C.-Y. Li C.-S. Fang S.-C. Wu C.-S. Liao D.-L. (2013). The reliability of the Chinese version of the Barratt impulsiveness scale version 11, in abstinent, opioid-dependent participants in Taiwan. *J. Chin. Med. Assoc.* 76 289–295. 10.1016/j.jcma.2013.01.005 23683263

[B50] Jha A. P. Stanley E. A. Kiyonaga A. Wong L. Gelfand L. (2010). Examining the protective effects of mindfulness training on working memory capacity and affective experience. *Emotion* 10 54–64. 10.1037/a0018438 20141302

[B51] Jo H. G. Schmidt S. Inacker E. Markowiak M. Hinterberger T. (2016). Meditation and attention: a controlled study on long-term meditators in behavioral performance and event-related potentials of attentional control. *Int. J. Psychophysiol.* 99 33–39. 10.1016/j.ijpsycho.2015.11.016 26659014

[B52] Kabat-Zinn J. (1990). *Full Catastrophe Living: Using the Wisdom of your Body and Mind to Face Stress, Pain, and Illness.* New York, NY: Delacorte Press.

[B53] Kabat-Zinn J. Massion A. O. Kristeller J. Peterson L. G. Fletcher K. E. Pbert L. (1992). Effectiveness of a meditation-based stress reduction program in the treatment of anxiety disorders. *Am. J. Psychiatry* 149 936–943. 10.1176/ajp.149.7.936 1609875

[B54] Kane M. J. Engle R. W. (2002). The role of prefrontal cortex in working-memory capacity, executive attention, and general fluid intelligence: an individual-differences perspective. *Psychon. Bull. Rev.* 9 637–671. 10.3758/BF03196323 12613671

[B55] Klumpp H. Angstadt M. Phan K. L. (2012). Insula reactivity and connectivity to anterior cingulate cortex when processing threat in generalized social anxiety disorder. *Biol. Psychol.* 89 273–276. 10.1016/j.biopsycho.2011.10.010 22027088 PMC3260042

[B56] Lee Y.-C. Chao H.-F. (2012). The role of active inhibitory control in psychological well-being and mindfulness. *Pers. Individ. Dif.* 53 618–621. 10.1016/j.paid.2012.05.001

[B57] Lipp O. V. (2006). “Human fear learning: contemporary procedures and measurement,” in *Fear and learning: From Basic Processes to Clinical Implications*, eds Craske M. G. Hermans D. Vansteenwegen D. (Washington, DC: American Psychological Association), 37–51. 10.1037/11474-002

[B58] Long D. L. Prat C. S. (2002). Working memory and stroop interference: an individual differences investigation. *Mem. Cogn.* 30 294–301. 10.3758/BF03195290 12035891

[B59] Luck S. J. Vogel E. K. (2013). Visual working memory capacity: from psychophysics and neurobiology to individual differences. *Trends Cogn. Sci.* 17 391–400. 10.1016/j.tics.2013.06.006 23850263 PMC3729738

[B60] Luck S. J. Vogel E. K. (1997). The capacity of visual working memory for features and conjunctions. *Nature* 390 279–281. 10.1038/36846 9384378

[B61] MacCallum R. C. Zhang S. Preacher K. J. Rucker D. D. (2002). On the practice of dichotomization of quantitative variables. *Psychol. Methods* 7 19–40. 10.1037/1082-989X.7.1.19 11928888

[B62] Manna A. Raffone A. Perrucci M. G. Nardo D. Ferretti A. Tartaro A. (2010). Neural correlates of focused attention and cognitive monitoring in meditation. *Brain Res. Bull.* 82 46–56. 10.1016/j.brainresbull.2010.03.001 20223285

[B63] Mayers A. (2013). *Introduction to Statistics and SPSS in Psychology.* London: Pearson.

[B64] McClelland G. H. Lynch J. G. Irwin J. R. Spiller S. A. Fitzsimons G. J. (2015). Median splits, Type II errors, and false–positive consumer psychology: don’t fight the power. *J. Consum. Psychol.* 25 679–689. 10.1016/j.jcps.2015.05.006

[B65] McLean C. P. Asnaani A. Litz B. T. Hofmann S. G. (2011). Gender differences in anxiety disorders: prevalence, course of illness, comorbidity and burden of illness. *J. Psychiatr. Res.* 45 1027–1035. 10.1016/j.jpsychires.2011.03.006 21439576 PMC3135672

[B66] Miyake A. Friedman N. P. (2012). The nature and organization of individual differences in executive functions: four general conclusions. *Curr. Dir. Psychol. Sci.* 21 8–14. 10.1177/0963721411429458 22773897 PMC3388901

[B67] Miyake A. Friedman N. P. Emerson M. J. Witzki A. H. Howerter A. Wager T. D. (2000). The unity and diversity of executive functions and their contributions to complex “Frontal Lobe” tasks: a latent variable analysis. *Cogn. Psychol.* 41 49–100. 10.1006/cogp.1999.0734 10945922

[B68] Mocaiber I. Pereira M. G. Erthal F. S. Figueira I. Machado-Pinheiro W. Cagy M. (2009). Regulation of negative emotions in high trait anxious individuals: an ERP study. *Psychol. Neurosci.* 2 211–217. 10.3922/j.psns.2009.2.014

[B69] Moore A. Malinowski P. (2009). Meditation, mindfulness and cognitive flexibility. *Conscious. Cogn.* 18 176–186. 10.1016/j.concog.2008.12.008 19181542

[B70] Moran T. P. (2016). Anxiety and working memory capacity: a meta-analysis and narrative review. *Psychol. Bull.* 142 831–864. 10.1037/bul0000051 26963369

[B71] Moreno A. L. Ávila-Souza J. Gomes W. B. Gauer G. (2015). Effects of worry on verbal and visual working memory. *Psychol. Neurosci.* 8 341–349. 10.1037/h0101277

[B72] Moriya J. (2016). Attentional networks and visuospatial working memory capacity in social anxiety. *Cogn. Emot.* 32 158–166. 10.1080/02699931.2016.1263601 27910724

[B73] Moriya J. Sugiura Y. (2012). High visual working memory capacity in trait social anxiety. *PLoS One* 7:e34244. 10.1371/journal.pone.0034244 22496783 PMC3322141

[B74] Moser J. S. Becker M. W. Moran T. P. (2012). Enhanced attentional capture in trait anxiety. *Emotion* 12 213–216. 10.1037/a0026156 22059521

[B75] Mrazek M. D. Franklin M. S. Phillips D. T. Baird B. Schooler J. W. (2013). Mindfulness training improves working memory capacity and GRE performance while reducing mind wandering. *Psychol. Sci.* 24 776–781. 10.1177/0956797612459659 23538911

[B76] Pacheco-Unguetti A. P. Acosta A. Callejas A. Lupiáñez J. (2010). Attention and Anxiety: different attentional functioning under state and trait anxiety. *Psychol. Sci.* 21 298–304. 10.1177/0956797609359624 20424060

[B77] Pashler H. (1988). Familiarity and visual change detection. *Percept. Psychophys.* 44 369–378. 10.3758/BF03210419 3226885

[B78] Patton J. H. Stanford M. S. Barratt E. S. (1995). Factor structure of the Barratt impulsiveness scale. *J. Clin. Psychol.* 51 768–774. 10.1002/1097-4679(199511)51:6<768::AID-JCLP2270510607>3.0.CO;2-1 8778124

[B79] Prinzmetal W. McCool C. Park S. (2005). Attention: reaction time and accuracy reveal different mechanisms. *J. Exp. Psychol. Gen.* 134 73–92. 10.1037/0096-3445.134.1.73 15702964

[B80] Quickel E. J. W. Johnson S. K. David Z. L. (2014). Trait mindfulness and cognitive task performance. *SAGE Open* 4:2158244014560557 10.1177/2158244014560557

[B81] Quirk G. J. Garcia R. Gonzalez-Lima F. (2006). Prefrontal mechanisms in extinction of conditioned fear. *Biol. Psychiatry* 60 337–343. 10.1016/j.biopsych.2006.03.010 16712801

[B82] Quirk G. J. Mueller D. (2008). Neural mechanisms of extinction learning and retrieval. *Neuropsychopharmacology* 33 56–72. 10.1038/sj.npp.1301555 17882236 PMC2668714

[B83] Rammstedt B. Danner D. Martin S. (2016). The association between personality and cognitive ability: going beyond simple effects. *J. Res. Pers.* 62 39–44. 10.1016/j.jrp.2016.03.005

[B84] Rouder J. N. Morey R. D. Morey C. C. Cowan N. (2011). How to measure working memory capacity in the change detection paradigm. *Psychon. Bull. Rev.* 18 324–330. 10.3758/s13423-011-0055-3 21331668 PMC3070885

[B85] Shek D. T. (1993). The Chinese version of the State-trait anxiety inventory: its relationship to different measures of psychological well-being. *J. Clin. Psychol.* 49 349–358. 10.1002/1097-4679(199305)49:3<349::AID-JCLP2270490308>3.0.CO;2-J 8315037

[B86] Spielberger C. D. Gorsuch R. L. Lushene R. E. (1970). *Manual for the State-Trait Anxiety Inventory.* Palo Alto, CA: Consulting Psychologists Press.

[B87] Srinivasan N. Singh A. (2017). Concentrative meditation influences visual awareness: a study with color afterimages. *Mindfulness* 8 17–26. 10.1007/s12671-015-0428-1

[B88] Stroop J. R. (1935). Studies of interference in serial verbal reactions. *J. Exp. Psychol.* 18 643–662. 10.1037/h0054651

[B89] Tabachnick B. G. Fidell L. S. (2007). *Experimental Designs using ANOVA.* Belmont, CA: Thomson / Brooks /Cole.

[B90] Tanaka K. Sugiura Y. Takebayashi Y. (2013). The relationship between orienting attention and dispositional mindfulness is moderated by alerting attention subtitle_in_Japanese. *Jpn. J. Pers.* 22 146–155. 10.2132/personality.22.146

[B91] Tang Y. Y. Lu Q. Fan M. Yang Y. Posner M. I. (2012). Mechanisms of white matter changes induced by meditation. *Proc. Natl. Acad. Sci. U.S.A.* 109 10570–10574. 10.1073/pnas.1207817109 22689998 PMC3387117

[B92] Tang Y. Y. Lu Q. Geng X. Stein E. A. Yang Y. Posner M. I. (2010). Short-term meditation induces white matter changes in the anterior cingulate. *Proc. Natl. Acad. Sci. U.S.A.* 107 15649–15652. 10.1073/pnas.1011043107 20713717 PMC2932577

[B93] Tang Y. Y. Ma Y. Wang J. Fan Y. Feng S. Lu Q. (2007). Short-term meditation training improves attention and self-regulation. *Proc. Natl. Acad. Sci. U.S.A.* 104 17152–17156. 10.1073/pnas.0707678104 17940025 PMC2040428

[B94] Tarrasch R. Margalit-Shalom L. Berger R. (2017). Enhancing visual perception and motor accuracy among school children through a mindfulness and compassion program. *Front. Psychol.* 8:281. 10.3389/fpsyg.2017.00281 28286492 PMC5323376

[B95] Teper R. Inzlicht M. (2013). Meditation, mindfulness and executive control: the importance of emotional acceptance and brain-based performance monitoring. *Soc. Cogn. Affect. Neurosci.* 8 85–92. 10.1093/scan/nss045 22507824 PMC3541488

[B96] Todd J. J. Marois R. (2004). Capacity limit of visual short-term memory in human posterior parietal cortex. *Nature* 428 751–754. 10.1038/nature02466 15085133

[B97] Unsworth N. Heitz R. P. Schrock J. C. Engle R. W. (2005). An automated version of the operation span task. *Behav. Res. Methods* 37 498–505. 10.3758/BF03192720 16405146

[B98] Vines L. M. (2014). *Dispositional Mindfulness and Working Memory in the Context of Acute Stress.* Doctoral dissertation, The University of Louisville’s Institutional, St, Louisville, KY.

[B99] Walsh J. J. Balint M. G. Smolira D. R. Fredericksen L. K. Madsen S. (2009). Predicting individual differences in mindfulness: the role of trait anxiety, attachment anxiety and attentional control. *Pers. Individ. Dif.* 46 94–99. 10.1016/j.paid.2008.09.008

[B100] Wang Z. Chen J. Boyd J. E. Zhang H. Jia X. Qiu J. (2011). Psychometric properties of the chinese version of the perceived stress scale in policewomen. *PLoS One* 6:e28610. 10.1371/journal.pone.0028610 22164311 PMC3229602

[B101] Way B. M. Creswell J. D. Eisenberger N. I. Lieberman M. D. (2010). Dispositional mindfulness and depressive symptomatology: correlations with limbic and self-referential neural activity during rest. *Emotion* 10 12–24. 10.1037/a0018312 20141298 PMC2868367

[B102] Wine J. (1971). Test anxiety and direction of attention. *Psychol. Bull.* 76 92–104. 10.1037/h0031332 4937878

[B103] Zalta A. K. Chambless D. L. (2012). Understanding gender differences in anxiety. *Psychol. Women Q.* 36 488–499. 10.1177/0361684312450004

[B104] Zeidan F. Johnson S. K. Diamond B. J. David Z. Goolkasian P. (2010). Mindfulness meditation improves cognition: evidence of brief mental training. *Conscious. Cogn.* 19 597–605. 10.1016/j.concog.2010.03.014 20363650

