# Corrigendum: Better Cognitive Performance Is Associated With the Combination of High Trait Mindfulness and Low Trait Anxiety

**DOI:** 10.3389/fpsyg.2018.01105

**Published:** 2018-06-26

**Authors:** Satish Jaiswal, Shao-Yang Tsai, Chi-Hung Juan, Wei-Kuang Liang, Neil G. Muggleton

**Affiliations:** ^1^Institute of Cognitive Neuroscience, National Central University, Taoyuan City, Taiwan; ^2^Institute of Cognitive Neuroscience, University College London, London, United Kingdom; ^3^Department of Psychology, Goldsmiths, University of London, London, United Kingdom

**Keywords:** personality traits, mindfulness, anxiety, executive functions, self-report measures

In the original article, the reference for Gaetano, 2018 was incorrectly written as Gaetano, J. (2018). *Holm-Bonferroni Sequential Correction: An Excel Calculator Microsoft Excel Workbook*. Available online at: www.staff.amu.edu.pl/~leka/_uploads/Holms-correction-calculator.xlsx. It should be Gaetano, J. (2018). *Holm-Bonferroni Sequential Correction: An Excel Calculator (v. 1.3) Microsoft Excel Workbook*. Available online at: https://www.researchgate.net/publication/322569220_Holm-Bonferroni_sequential_correction_An_Excel_calculator_13. The authors apologize for this error and state that this does not change the scientific conclusions of the article in any way.

The original article has been updated.

## Conflict of interest statement

The authors declare that the research was conducted in the absence of any commercial or financial relationships that could be construed as a potential conflict of interest.

